# Radiotherapy as a Single Modality in Primary Seminoma of the Prostate

**DOI:** 10.7759/cureus.14264

**Published:** 2021-04-02

**Authors:** Aquila Akingbade, Darin Gopaul, Harry C Brastianos, Stacey Hubay

**Affiliations:** 1 Radiation Oncology, Queen's University, Kingston, CAN; 2 Radiation Oncology, Grand River Regional Cancer Centre, Kitchener, CAN; 3 Medical Oncology, Grand River Regional Cancer Centre, Kitchener, CAN

**Keywords:** radiotherapy (rt), prostate seminoma, prostate cancer, extragonadal germ cell tumor

## Abstract

Extragonadal germ cell tumors (EGCTs) are uncommon, and those involving the prostate are rare. We report on a primary seminoma of the prostate in a 56-year-old male presenting with scrotal pain, urinary frequency and urgency, and erectile dysfunction. Digital rectal examination revealed a hard, markedly enlarged prostate projecting posteriorly into the rectum. All 12 cores from ultrasound-guided prostate biopsy revealed malignant cells that stained positive for OCT4, PLAP, and CD117. Imaging revealed a 10.2 cm x 7.8 cm x 8.4 cm prostate mass with irregular nodular margins extending superiorly to the base of the bladder and posteriorly abutting the anterior rectal wall. There was no evidence of distant metastatic disease on both nuclear medicine and CT scans of the chest, abdomen, and pelvis. An 11 mm right internal iliac lymph node and several tiny sub-centimeter external iliac nodes were noted bilaterally. The patient was treated with radiotherapy to the prostate and pelvic lymph nodes. The pelvic lymph nodes were treated with 20 Gy in eight fractions, followed by a boost to the prostate for a further 20 Gy in eight fractions. There was a significant response during treatment that allowed an adaptive boost for a further 10 Gy in four fractions to bring the total dose to the prostate to 50 Gy in 20 fractions. Treatment was well tolerated. Adjuvant chemotherapy was not recommended. He remains disease-free 24 months post-treatment. This case report indicates that like most seminomas, extragonadal seminomas are exquisitely sensitive to radiotherapy and may be considered for the primary treatment of non-metastatic disease. To our knowledge, this is the first reported case of the sole use of radiotherapy to treat a primary seminoma of the prostate.

## Introduction

Extragonadal germ cell tumors (EGCTs) are uncommon entities that typically originate in the midline of the body, such as the mediastinum, retroperitoneum, pineal gland, and sacrococcygeal areas. EGCTs are divided into seminomatous tumors, consisting only of seminomas, and non-seminomatous tumors, consisting of choriocarcinomas, teratomas, yolk sac carcinomas, and mixed tumors [1]. EGCTs of the prostate are rare, and to date, there are only 10 instances of seminoma of the prostate in the literature [2-11]. In this case report, we present a patient with primary seminoma of the prostate, treated with radiotherapy only. Patient consent was obtained for chart review and case report.

## Case presentation

Clinical findings

A 56-year-old male presented to his family physician with scrotal pain and erectile dysfunction of three months duration. This was associated with increased urinary frequency and urgency. There was also an associated 20-pound weight loss over that period. Past medical history is remarkable for stage 1 left testicular seminoma treated with a radical inguinal orchiectomy and no adjuvant or neoadjuvant treatment 30 years earlier. Ultrasound of the contralateral right testicle was unremarkable, and urology was consulted.

Digital rectal examination revealed a markedly enlarged and hard prostate projecting posteriorly into the rectum. A bladder scan demonstrated a post-void residual volume of 279 mL. He was started on Flomax CR (tamsulosin) 0.4 mg po q.h.s. without improvement.

Serum beta-human chorionic gonadotropin (b-hCG) and lactose dehydrogenase (LDH) levels were elevated at 6 IU/L and 628 IU/L, respectively, while serum alpha-fetoproteins (AFP) and prostate-specific antigen (PSA) were normal at 2 µg/L and 1 µg/L, respectively.

**Figure 1:**
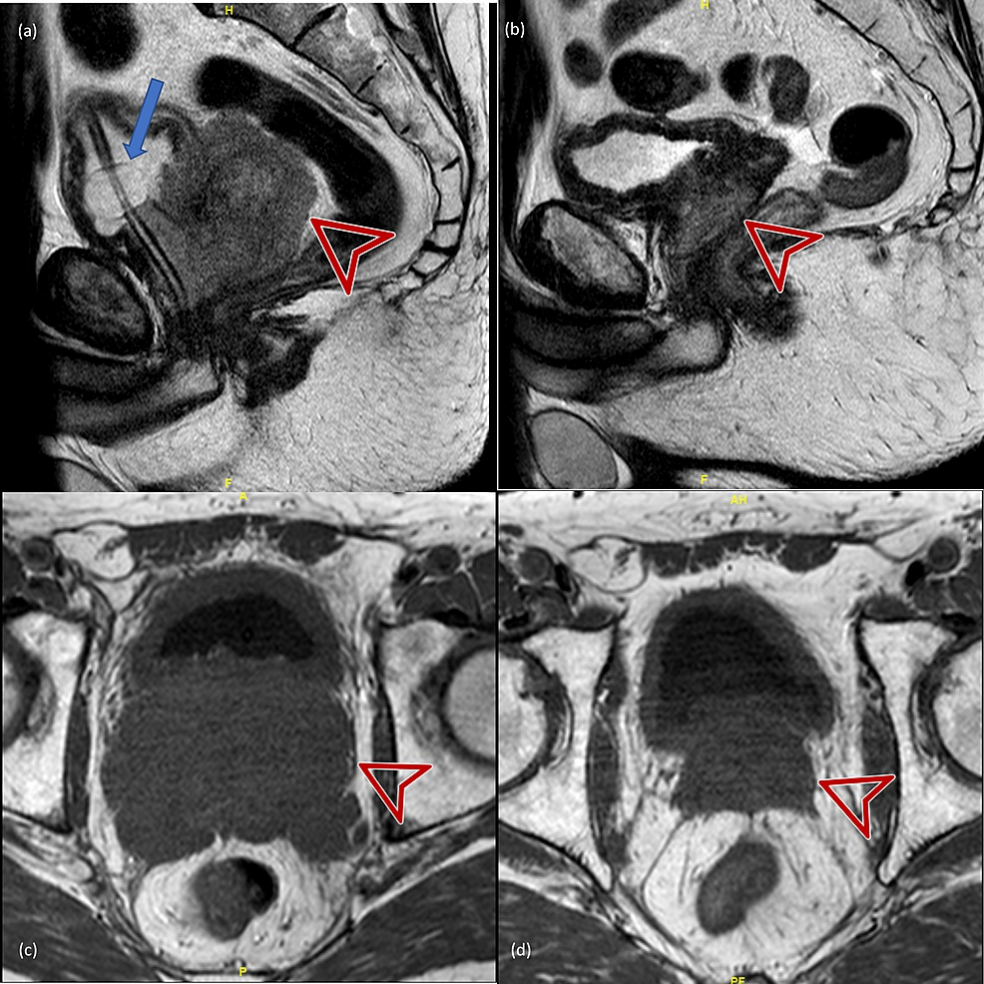
Pre-treatment vs. four-month post-treatment MRI scans. (a, c) T2 MRI scan pre-treatment showing a large heterogeneous prostate (red arrows). (b, d) T2 MRI showing a normal-appearing prostate (red arrows) four months after delivery of 50 Gy in 20 fractions. Top row = Sagittal view, bottom row = Axial view. The blue arrow points toward a Foley catheter.

CT of the abdomen and pelvis demonstrated diffuse heterogenous prostatic enlargement and enhancement (Figure 1a). There was no reported involvement of the pelvic or retroperitoneal lymph nodes on CT. There was no evidence of distant metastases. Bone scan demonstrated no evidence of bony metastases.

One month later, his urinary symptoms progressed with decreased urinary flow, nocturia eight times per night, suprapubic discomfort, and increased difficulty with bowel movements. A follow-up bladder scan demonstrated a post-void residual volume of 605 mL. A Foley catheter was placed.

MRI of the pelvis revealed marked diffuse enlargement of the entire prostate with irregular nodular margins and extraprostatic extension along the left and right lateral pelvic walls with small, soft tissue nodules along the left lateral pelvic wall and possible tiny nodularity on the right lateral pelvic wall. The prostatic mass extended superiorly into the base of the bladder, abutted the anterior rectal wall and levator ani posteriorly and inferiorly, respectively. The prostate measured 10.2 cm x 7.8 cm (axial) and 8.4 cm (cranial-caudal). The seminal vesicles were completely replaced by the prostatic mass. There was an 11 mm enhancing right internal iliac lymph node. Several tiny sub-centimeter bilateral external iliac nodes and a few tiny sub-centimeter perirectal nodes were noted. He was staged as T4 N1 M0 (Stage IVa) according to the AJCC 8th edition for prostate cancer.

Pathologic and immunohistochemical findings

A transrectal ultrasound-guided prostate biopsy was performed. Pathology reported involvement of all 12 cores with malignant cells arranged in sheets without gland formation. The malignant cells had large nuclei, clear cytoplasm, and prominent nucleoli. Immunohistochemistry demonstrated: OCT4+, PLAP+, CD117+, PSA-, PSAP-, S100-, LCA-, CT20-, CB3-, and PAX5-. The pathologic diagnosis was seminoma. No prostatic carcinoma was identified.

Treatment and results

The case was reviewed at our regional Genitourinary Tumor Board (multidisciplinary cancer conference) consisting of at least one representative from diagnostic radiology, medical oncology, urology, radiation oncology, and pathology. The consensus was that this was a primary seminoma of the prostate that was limited to the pelvis. While the 1.1 cm right internal iliac lymph node was suspicious for involvement, there was no evidence of para-aortic lymphadenopathy or distant metastasis. His disease was deemed unresectable. Seminomas were noted to be sensitive to both chemotherapy and radiotherapy. In the absence of distant metastasis, primary radiotherapy was recommended. 

CT simulation and radiation planning were performed. He was treated with hypofractionated volumetric-modulated arc therapy (VMAT) radiotherapy in three sequential phases. Phase one consisted of radiotherapy to the prostate and pelvic lymph nodes with a dose of 20 Gy in eight daily 2.5 Gy fractions. Phase two consisted of a boost to the prostate with a further dose of 20 Gy in 8 fractions. The gross tumor volume (GTV) was measured at 334 cm^3^. CT simulation was repeated at the start of the third week of radiotherapy and a significant response was observed. The GTV now measured 138 cm^3^. He was re-planned and treated with a phase three reduced field size (adaptive) boost for a further 10 Gy in four fractions to the residual prostate mass. The total dose to the prostate was 50 Gy in 20 fractions in over four weeks.

The treatment was well tolerated. The patient experienced mild treatment-related diarrhea which was managed by dietary modification (Grade 1, based on Radiation Therapy Oncology Group [RTOG] acute radiation toxicity grading). Following completion of radiotherapy, his lower gastrointestinal symptoms resolved. His Foley catheter was removed, with no residual urinary symptoms at a one-month follow-up. Table 1 shows the evolution of tumor markers following treatment.

**Table 1:** Evolution of serum markers. AFP: Alpha-fetoprotein; b-hCG: Beta-human chorionic gonadotropin; LDH: Lactose dehydrogenase; PSA: Prostate-specific antigen; PT: Post-treatment.

Serum marker (range of normal)	Pre-treatment	1-month PT	4-months PT	7-months PT	12-months PT	24-months PT
AFP (0-10 µg/L)	2	2	2	2	2	2
b-hCG (0-4.99 IU/L)	6	<2	<2	<2	<2	<2
PSA (0-4 µg/L)	0.69	1.0	0.38	0.34	0.43	-
LDH (313-613 IU/L)	628	-	-	-	-	546

Follow-up MRI four months post-treatment (Figures 1b, d) confirmed a significant decrease of the prostate mass from 10 cm x 7.8 cm x 7.4 cm to 4.9 cm x 3.5 cm x 4 cm. There were no discrete residual masses or enhancing lesions within the prostate, consistent with a complete response.

Post-treatment surveillance includes clinical follow-up, CT chest abdomen, and pelvis and pelvic MRI every 3-4 months for the first two years, then CT chest abdomen and pelvis every six months for the third year, then annually until five years post-treatment.

He remains alive and well. There is no late urinary or rectal toxicity, and he is free of recurrent disease on CT and MRI at last follow-up 24 months post-treatment.

## Discussion

The primary seminoma of the prostate is a rare entity. This patient has a history of testicular seminoma with a left orchiectomy thirty years prior. Most recurrences of testicular germ cell tumors occur within two years of treatment. While the longest recurrence for a germ cell tumor is 43 years [12], late recurrences (>2 years post-treatment) are uncommon; the relapse-free rates in 725 patients with stage 1 testicular seminoma managed with surveillance, radiotherapy, 1x carboplatin, 2x carboplatin were 91.8%, 97.6%, 95%, and 98.5%, respectively, after 30-month follow-up. The recurrence pattern for testicular seminomas is predictable as most occur in retroperitoneal sites, followed by the lung and mediastinal sites, then neck and supraclavicular sites [13]. As such, we believe this presentation is a primary prostatic seminoma.

There is a paucity of data to guide the selection of radiotherapy doses for prostate seminoma. Testicular seminomas tend to be radiosensitive and typical non-adjuvant doses are 35-40 Gy [14, 15]. Accelerated hypofractionation with 20 fractions is a standard approach at our center for pelvic malignancies and was thought to be optimal in this setting due to the reduced treatment time and well-established dose-volume toxicity criteria for the adjacent bowel and bladder. We had hoped the reduced treatment time would reduce exposure to the adjacent normal tissue given the anticipated tumor response, as demonstrated on the intra-treatment repeat CT simulation for the adaptive boost.

Due to the rarity of prostatic seminomas, our search strategy involved the selection of PubMed-indexed publications of primary prostatic seminomas, and the exclusion of reports of metastatic disease to the prostate. To our knowledge, there are only ten reported cases of prostate seminoma in the medical literature. Of the ten cases, eight were localized to the pelvis while the others had retroperitoneal involvement [3] or metastatic disease [4]. Six of the eight cases with pelvis-localized disease provided information on treatment and outcome data and these are summarized in Table 2. Of the two case reports without outcome data, one was a vignette on 18-fluoro-2-deoxyglucose positron emission tomography (PET) in a case of prostate seminoma [5], while the other halted treatment after the patient developed a myocardial infarction after two cycles of bleomycin, etoposide, and cisplatin (BEP) chemotherapy [11]. 

**Table 2:** Summary of reported localized prostatic seminomas. AFP: Alpha-fetoprotein; HCG: Human chorionic gonadotropin; OCT: Octamer motif; PAS: Periodic acid-Schiff stain; PLAP: Placental alkaline phosphatase; PSA: Prostate-specific antigen; PSAP: Prostate-specific alkaline phosphatase; TURP: Transurethral resection of the prostate.

First Author, Year of Publication	Age at diagnosis	Clinical presentation	Immunohistochemistry	Diagnosis	Therapy	Outcomes
Khandekar, 1993 [6]	58	Gross hematuria, urgency, burning on urination	PLAP + PAS + Cytokeratin - Vimentin +	Seminoma of the prostate with bladder neck involvement	Chemotherapy (two courses of bleomycin, cisplatin, etoposide)	Complete remission at 10 months
Hayman, 1995 [7]	31	Hemospermia, hematuria, dysuria, terminal dribbling	PLAP + Cytokeratin + PSA -	Prostatic seminoma involving bladder base, seminal vesicles, and right iliac lymph nodes	Chemotherapy (four courses of bleomycin, etoposide, cisplatin). Adjuvant radiotherapy (40 Gy in 20 fractions)	Complete remission at 13 years
Han, 2003 [2]	24	Continuous lumbago, macrohematuria	Seminoma portion: PLAP + PAS - AFP + HCG - Cytokeratin - Vimentin - PSA -	Primary EST (yolk sac tumor) with focal seminoma of the prostate with bladder neck involvement	Surgery (radical prostatectomy), Chemotherapy (four courses of cisplatin, peplomycin, doxorubicin, mitomycin C, cyclophosphamide, dacarbazine, etoposide)	Died at eight months due to lung, liver, and brain metastases of the EST portion
Hashimoto, 2009 [9]	54	Dysuria	PLAP + PAS + CD117 + AE1/AE3 + Cytokeratin - Vimentin - PSA -	Seminoma of the prostate with bladder wall involvement	Chemotherapy (three courses of bleomycin, cisplatin, etoposide)	Complete remission at 28 months
Zheng, 2015 [8]	54	Dysuria, nocturia, nocturnal incontinence	-N/A-	Primary prostate seminoma with bladder and lymph node involvement	Surgery (pelvic exenteration, orchiectomy), chemotherapy (nine cycles of cyclophosphamide)	Complete remission at 11 years
Kazmi, 2019 [10]	54	Frequency, urgency, poor stream	CD117+ PLAP+ PSA- Cytokeratin- CAM1- AE1/AE3-	Primary seminoma of the prostate	TURP, Chemotherapy (four cycles of bleomycin, etoposide, platinum)	Complete remission at four years
Akingbade, 2020 (current case)	56	Scrotal pain, erectile dysfunction, increased urinary frequency, and urgency	PLAP+ OCT4+ CD117+ PSA-	Primary seminoma of the prostate involving bladder base, seminal vesicles, and iliac lymph nodes	Radiotherapy (50 Gy in 20 fractions)	Complete remission at 24 months

Similar to our case, five of the six cases with outcome data noted invasion of adjacent structures (seminal vesicles and bladder base) [2, 6-9]. Of the six, two were treated with chemotherapy only [6, 9], one was treated with chemotherapy followed by radiotherapy [7], and three were treated with a combination of surgery and chemotherapy [2, 8, 10]. Of the three patients who received surgery, one was treated with radical prostatectomy, another with pelvic exenteration, and the last with TURP. All six patients had complete remission.

With an average follow-up of 55.4 months (range 5-156 months), there was one reported death [2-4, 6-10]. This patient was diagnosed with a yolk sac tumor with focal seminoma of the prostate and involvement of the bladder neck. He was treated with radical prostatectomy and four cycles of chemotherapy but died of metastatic disease of the yolk sac component to the lungs, liver, and brain eight months later [2].

## Conclusions

A complete response was obtained with radiotherapy as a single modality in this patient with a bulky seminoma of the prostate and a suspicious pelvic lymph node. There was no evidence of recurrence at 24 months. To the best of our knowledge, our case report is the first instance of the sole use of radiotherapy for primary prostatic seminoma in the literature, and we present single modality radiotherapy as a consideration for the treatment of localized, non-metastatic disease.
